# Formyl Peptide Receptor as a Novel Therapeutic Target for Anxiety-Related Disorders

**DOI:** 10.1371/journal.pone.0114626

**Published:** 2014-12-17

**Authors:** Irene Gallo, Lorenza Rattazzi, Giuseppa Piras, Thomas Gobbetti, Elisabetta Panza, Mauro Perretti, Jeffrey W. Dalley, Fulvio D'Acquisto

**Affiliations:** 1 William Harvey Research Institute, Barts and the London School of Medicine, Queen Mary University of London, London, United Kingdom; 2 Department of Experimental Pharmacology, University of Naples “Federico II”, Naples, Italy; 3 Department of Psychology, University of Cambridge, Cambridge, United Kingdom; 4 Department of Psychiatry, University of Cambridge, Cambridge, United Kingdom; University of Insubria, Italy

## Abstract

Formyl peptide receptors (FPR) belong to a family of sensors of the immune system that detect microbe-associated molecules and inform various cellular and sensorial mechanisms to the presence of pathogens in the host. Here we demonstrate that Fpr2/3-deficient mice show a distinct profile of behaviour characterised by reduced anxiety in the marble burying and light-dark box paradigms, increased exploratory behaviour in an open-field, together with superior performance on a novel object recognition test. Pharmacological blockade with a formyl peptide receptor antagonist, Boc2, in wild type mice reproduced most of the behavioural changes observed in the *Fpr2/3* ^-/-^ mice, including a significant improvement in novel object discrimination and reduced anxiety in a light/dark shuttle test. These effects were associated with reduced FPR signalling in the gut as shown by the significant reduction in the levels of p-p38. Collectively, these findings suggest that homeostatic FPR signalling exerts a modulatory effect on anxiety-like behaviours. These findings thus suggest that therapies targeting FPRs may be a novel approach to ameliorate behavioural abnormalities present in neuropsychiatric disorders at the cognitive-emotional interface.

## Introduction

The immune system is equipped with a vast variety of biological weapons to sense the presence of pathogens *via* the recognition of pathogen-associated molecular patterns (PAMPs) [1], [2]; these elicit a complex series of events leading to the specialization and differentiation of the immune cells, B and T lymphocytes [3]. Formyl peptide receptors (FPRs) are G protein-coupled receptors whose main function is to sense the presence of harmful or noxious molecules such as formylated peptides and guide cells to the site where pathogen-associated molecules have been released [4]. This sensing function of FPRs is not limited to a particular pathogen and is extended to a wide range of endogenous ligands including classical biomarkers of inflammation and immune activation such as serum amyloid A (SAA) [5], formylated peptides released by mithochondria of damaged cells and tissue [6], the antimicrobial peptide LL-37 [7] and the dual pro- and anti-inflammatory protein Annexin-A1 [8].

There are currently three functional FPRs in humans as well as in mouse - FPR1, FPR2 and FPR3- which all recognise to different degrees a wide range of endogenous and exogenous ligands [6], [9], [10]. Activation of these receptors causes their homo- or hetero-dimerization which in turn depends on the precise ligand they bind to [11], [12]. In this way FPRs are able to exert both pro- and anti-inflammatory effects on immune cells [4], [8], [10].

The expression of FPRs is highest in sentinel innate cells with phagocytic or chemotactic activity such as neutrophils [13], [14], monocytes [13], [15], macrophages[15], [16] and dendritic cells [15], [17]. However, FPR are also expressed in non-phagocytic and “immobile” sentinel cells such as mucosal epithelial cells [18], [19], endothelial cells [20]–[22] and glia [23]–[25]. In these cells, FPRs exert a genuine “sentinel role” by sensing pathogens present in the *microenvironment* as well as by favouring repair upon damage and inflammation. Recent findings show that FPRs are expressed in the vomeronasal system, where they are postulated to detect the presence of infection in the “macro environment” through volatile FPR ligands present in the faeces of pathogen-infected animals [26]–[29]. Thus, FPRs exert a unique role in the response of the host to pathogens because they signal at two levels; firstly at the level of the central nervous system to alert the host of impeding dangers and secondly at the level of the immune system by initiating a protective inflammatory response.

Recent findings indicate that the centrally regulated behaviours of anxiety and fear-elicited responses are strongly modulated by FPR1 [30]. These data suggest that FPRs may play a permissive role in the pathophysiology of various psychiatric disorders, which increasingly implicate immunological mechanisms in their aetiology [31]–[33]. In the present study we investigated various anxiety-related behaviours in *Fpr2/3* ^-/-^ mice [34], including responses to novelty and aversive contextual stimuli, and compared the selectivity of these responses with low anxiety-provoking behaviours. We report that *Fpr2/3* ^-/-^ mice show increased explorative behaviour and reduced fear compared with wild type littermates. Notably, the behavioural profile of *Fpr2/3* ^-/-^ mice was partially mimicked by intraperitoneal injection of the pan-FPR inhibitor Boc2 [35], [36], which was accompanied by a decreased activation of downstream FPR signalling pathways in the gut. Together these results support the hypothesis that FPRs may have an important role to play in the regulation of aversive emotional responses. Thus targeting FPRs might provide new avenues of treatment for a range of brain disorders linked to anxiety.

## Materials And Methods

### Mice

Four to six week old male mice were used for all experiments. *Fpr2/3* ^-/-^ mice have been previously described [34] and were backcrossed onto C57BL/6 for more than 8 generations. Animals were housed in groups of 4–5 under specific-pathogen-free conditions, with free access to food and water and in a room under a 12 h light/dark cycle (light on at 7:00 am). C57BL/6 mice were purchased from Charles River (Margate, UK) and housed for at least 10 days in the same room as the *Fpr2/3* ^-/-^ prior to testing to allow acclimatization. *Fpr2/3* ^+/+^ littermate controls and C57BL/6 mice were used in equal number and are collectively referred to as wild-type controls since they showed no significant differences in all the preliminary tests. All animal studies were conducted with ethical approval from the Local Ethical Review Committee. This research was carried out in accordance with the UK Animals (Scientific Procedures) Act, 1986 and under the UK Home Office project license number 70/6994.

### Behavioural tests and pharmacological treatment

If not otherwise stated, tests were performed double-blind every other day during the light phase of the light-dark cycle, as previously described and recommended [37]. All the efforts were made to minimize mouse discomfort in these behavioral experiments. Mice were brought to the testing room at least 30 minutes before the start of the test session to allow habituation to the testing environment. Unless otherwise specified, standard lighting (about 50 lux) and quiet conditions were maintained throughout each experiment. FPR antagonist studies were performed with male C57BL/6 mice receiving an intraperitoneal injection of the FPR2 antagonist Boc2 (t-Boc-FLFLF; at a previously validated dose of 10 µg/animal [38], [39] or an equal volume of phosphate-buffered saline (PBS) as a control solution (200 µl), 30 minutes before the behavioural tests. This research was carried out in accordance with the UK Animals (Scientific Procedures) Act, 1986.

### Open field activity test

**Figure 1:**
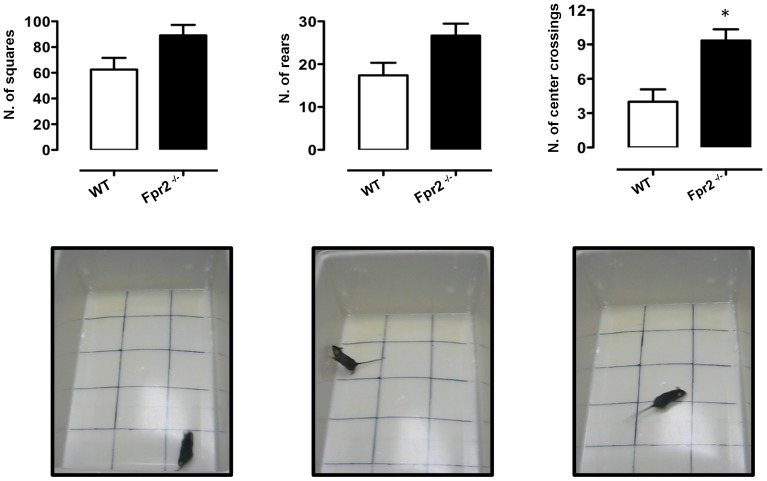
Reduced anxiety-like behaviour of *Fpr2/3* ^-/-^ mice in the open field test. The bar graphs and images show total number of squares crossed, rears and centre crossings during a 5-minute session. Values are expressed as median ± S.E.M. and representative of four experiments, each involving 6–9 mice per group. * *P*<0.05 indicate significant values compared with wild-type (WT) control mice (Mann–Whitney *U*-test).

The open field test (OFT) is an ethologically based paradigm that provides objective measures of exploratory behaviour as well as a valid initial screen for anxiety-related behaviour in rodents and was carried out as previously described with some modifications [40]. The apparatus consisted of a white PVC arena (50 cm×30 cm×20 cm) divided into 10 cm×10 cm squares (n = 15). The 3 central squares defined the “centre” region (see Fig. 1). Each mouse was placed in a corner square, facing the wall, and observed and recorded for 3 minutes. The total number of squares crossed (all four paws in), total number of rears (defined as both front paws off the ground, but not as a part of grooming) and number of centre crossings was recorded. The walls and floor of the arena were thoroughly cleaned between each trial.

### Climbing activity test

**Figure 2:**
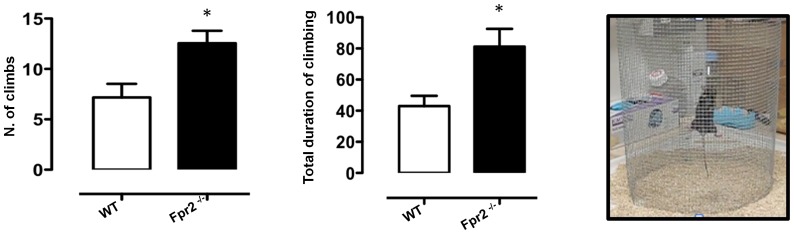
Increased exploratory behaviour of *Fpr2/3* ^-/-^ mice in the climbing test. The bar graphs show the number of climbing events and total time (seconds) spent on the climbing mesh during a 5-minute trial. Values are expressed as median ± S.E.M. and representative of three different experiments, each involving 6–9 mice per group. * *P*<0.05 indicate significant values compared with wild-type (WT) control mice (Mann–Whitney *U*-test).

The climbing test is used to assess vertical activity and exploratory behaviour. The test was performed as previously described but with some modifications [41], [42]. Briefly, mice were placed, one at a time, on a thin layer of fresh wood chip bedding on a laboratory bench and covered with a cylindrical climbing mesh (60 cm×30 cm base diameter) (see Fig. 2). They were each observed and recorded for 5 minutes. The number of climbing events and total duration of climbing activity was assessed. The criterion for climbing was for a mouse to have all 4 feet on the wire mesh while a climb terminated as soon as one foot touched the bench. This test was conducted in the late afternoon, when mice are known to be more active [43].

### Light-dark shuttle box

**Figure 3:**
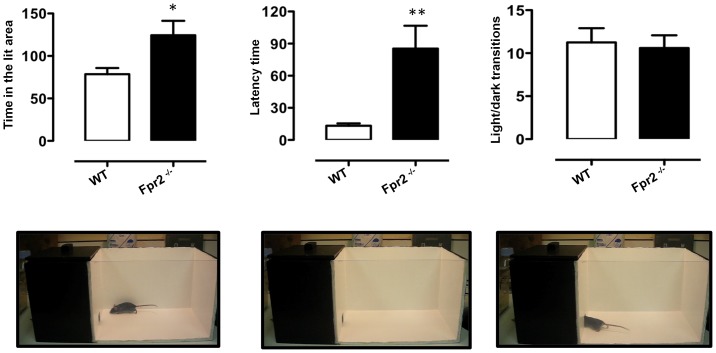
Reduced anxiety-like behaviour of *Fpr2/3* ^-/-^ mice in the light and dark box test. The bar graphs and images show the total time (seconds) spent in the lit area, latency (seconds) to first cross to the dark chamber and total number of transition during a 5-minute trial. Values are expressed as median ± S.E.M. and representative of four different experiments involving 6–9 mice per group. * *P*<0.05 and ** *P*<0.05 indicate significant values compared with wild-type (WT) control mice (Mann–Whitney *U*-test).

In this test exploratory activity reflects the combination of hazard and risk avoidance [44]. The apparatus consisted of a 45 cm×20 cm×21 cm box, divided into two distinct compartments: one third (15 cm long) painted black, with a black lid on top, the remaining two thirds painted white and uncovered (see Fig. 3). A 2.5 cm×2.5 cm opening joined the two compartments. One side of the bright box was transparent to enable behavioural assessment and the averseness of this compartment was increased by additional illumination supplied by a 50 W lamp placed 45 cm above the centre of the box floor. The test was performed in accordance with a previous published protocol [45]. Each mouse was placed in the bright compartment, facing away from the opening and allowed to explore the box for 5 minutes. Dependent variables included the time spent in the light area, latency to cross to the dark area (all four paws in) and the total number of transitions between compartments. The apparatus was cleaned after each trial.

### Marble burying test

**Figure 4:**
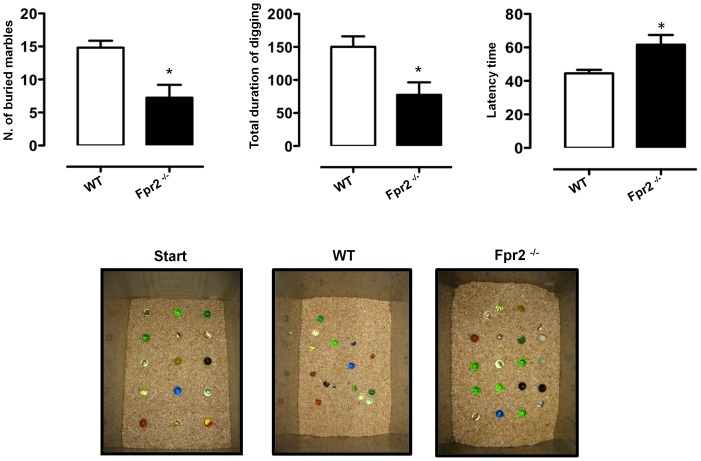
Reduced digging and marble burying behaviour of *Fpr2/3* ^-/-^ mice in the marble burying test. The bar graphs and relative pictures show the total number of buried marbles, total duration (seconds) of digging and the latency (seconds) to the first digging bout during a 10-minute trial. Values are expressed as median ± S.E.M. and representative of four different experiments involving 6–9 mice per group. * *P*<0.05 and ** *P*<0.05 indicate significant values compared with wild-type (WT) control mice (Mann–Whitney *U*-test).

The marble-burying test (MBT) is thought to reflect repetitive and perseverative behaviour, possibly related to compulsions and/or anxiety disorders [46]. The test was carried out as described by Deacon and colleagues [47] with some modifications. Briefly, mice were individually placed in a clear plastic box (14 cm×10 cm×11 cm) filled with approximately 5 cm depth of wood chip bedding lightly pressed to give a flat surface (see Fig. 4). Fifteen 1.5 cm diameter glass marbles were placed on the surface, evenly spaced, each about 4 cm apart, so to form 5 rows of 3. The latency to start digging (defined as the mouse digging the bedding with front and hind paws for more than 1 second), the total number of digging bouts and the number of buried marbles (to 2/3 of their depth) were manually recorded during the 10 minute-test.

### Novel object recognition test (NORT)

The novel object recognition test has been widely used to assess the mouse's ability to discriminate between a previously encountered and a novel object [48]. The test relies on the idea that mice approach more frequently and spend more time exploring a novel object when previously exposed to a familiar one that they recognize as already encountered. The test was carried out as previously described [49], [50]. On day one mice were firstly habituated to the open-field box for 10 minutes. On day two, mice were placed in the same arena for a 10 minute acquisition period, during which time they were exposed to two identical toys (3 cm^3^ non-toxic red wooden cubes (object A and B). Objects were glued to the floor 10 cm apart from each other, 8 cm away from both box edges. After being returned in their home cage, mice were given a one-hour inter-trial interval. Each subject was then placed back into the arena, where everything was the same as during the acquisition phase except that object A was replaced with a wooden, green, cylinder (4 cm height, 1 cm base diameter) (novel object). During this test phase, mice were allowed to explore both of the objects for 5 minutes. Acquisition and test phases were recorded with a video camera and time spent visiting each object (visit defined as when the animal's nose touched the object or was pointed towards it within 1 cm radius) was manually assessed in both phases. Results were expressed as percentage of object discrimination [(Time spent exploring novel object/total time spent exploring during testing phase) ×100]. After each trial both the arena and the objects were cleaned with 70% ethanol, in order to eliminate olfactory traces.

### Y-Maze spontaneous alternation test

The Y maze was made of three enclosed transparent plastic arms (A, B, C) 29 cm×8 cm×19 cm each, set at an angle of 120° to each other in the shape of a Y. It was fixed on a white wooden board and placed on the floor of a room containing several large immovable objects to use as spatial cues. In this test for spatial memory mice tend to enter the maze arm that was explored most recently and remember the order of the arm entry, thanks to their ability to allocate the arm's positions through spatial clues surrounding the testing apparatus. Mice were allowed to freely explore the arena for 5 minutes, during which time the total number of arm entries was recorded, along with the entering sequence, not including the initial arm. A spontaneous alternation occurred when the animal entered into all three arms of the maze on consecutive choices in overlapping triplet sets (e.g. CBABCABCBACB  = 8 alternations) [51]. Spontaneous alternation percentage was calculated as: [Total number of actual alternations/(total arm entries –2)] ×100. The maze was thoroughly cleaned after each test.

### Colon whole mount preparation

Colon biopsies were washed with PBS and fixed in 4% paraformaldehyde (PFA). Samples were washed 2 times with PBS and then permeabilised with PBS containing 0.1% Triton X-100 for 5 minutes. Thereafter, samples were washed again, then blocked in PBS containing 5% foetal bovine serum (FBS) for 1 hour. Samples were incubated alternatively with mouse monoclonal anti-phospho-p38 (#sc-7973, Santa Cruz Biotechnology) (1∶100 dilutions) for 90 minutes and then all with Alexa Fluor 488 goat polyclonal anti-mouse IgG (H+L) (ab150113, Abcam) 1∶100 for 1 hour. Rinsed samples were finally mounted in Optimum Cutting Temperature (O.C.T.; Tissue-Tek) and frozen at −80°C. Five µm thick sections were mounted on slides and visualized by fluorescence microscopy [52].

### Plasma corticosterone and cytokine measurement

Blood was collected from untested mice by intracardiac puncture performed under anaesthesia, and all efforts were made to minimize suffering. Plasma was obtained from the clotted blood by centrifugation (8000 rpm, 5 min) and stored at −80°C before the assay. Corticosterone concentrations were measured in diluted (1∶32) plasma by Enzymatic Immuno Essay (EIA) assay following the manufacturer's instructions (Enzo Life Sciences, Exeter, UK). Cytokine levels in the same samples were measured (dil. 1∶500) using mouse Th1/Th2/Th17/Th22 16 plex Kit FlowCytomix and according to the manufacturer's instructions (eBioscience).

### Statistical analysis

Results were analysed as previously described [53]–[55] using GraphPad. Unpaired Student's *t* test was performed for experiments where differences between two groups needed to be analysed. For non-parametric data, the Mann–Whitney *U*-test was applied and results were expressed as medians (interquartile range). Statistical significance was determined at *p*<0.05. The results were expressed as mean ± S.E.M.

## Results

### Reduced anxiety in *Fpr2/3* null mice

Although we found no statistically significant difference between *Fpr2/3* ^-/-^ mice and wild-type control mice with respect to ambulation and rearing (**Fig. 1**, **left** and **middle panels**, respectively), *Fpr2/3* ^-/-^ mice showed reduced thigmotaxis (walking along the edges) and significantly increased centre crossings (Fig. 1, right panel) indicating a reduced level of anxiety [56], [57].

We further tested anxiety-related behaviour using the climbing test where vertical exploratory behaviour is assessed [41], [42]. As shown in **Fig. 2**, *Fpr2/3* ^-/-^ mice performed a greater number of climbing acts compared with wild-type (p<0.05) and spent on average more time climbing than control animals (p<0.05).

We next investigated anxiety behaviour using the light/dark shuttle box and the marble-burying test. Consistent with our earlier results, *Fpr2/3* ^-/-^ mice spent significantly more time in the aversive, brightly lit compartment compared with wild-type controls (p<0.05) and waited longer to move to the less aversive, dark side of the box (p<0.01) (**Fig. 3**, **left** and **middle panels**, respectively).

*Fpr2/3* ^-/-^ mice also buried less marbles and spent less time in this activity compared with wild-type (**Fig. 4** **left** and **middle panels**, respectively). The latency to start this behaviour was also significantly increased in *Fpr2/3* ^-/-^ mice (**Fig. 4** right panel) consistent with reduced anxiety.

### Improved novel object recognition in *Fpr2/3* ^-/-^ mice

**Figure 5:**
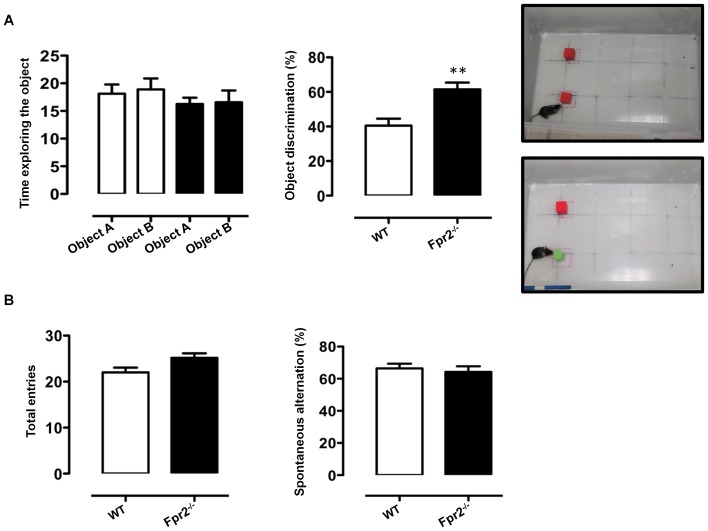
Increased discriminatory activity of *Fpr2/3* ^-/-^ mice in the novel object recognition test. The bar graph in **A** shows the total time (seconds) spent exploring the objects used in the test (shown in the top picture) during the 10-minute acquisition phase (left panel) and the % of time spent on the novel object (shown in the bottom picture) in the subsequent 5-minute test phase (right panel). The bar graphs in **B** show the total number of arm entries and spontaneous alternation percentage (calculated as described in material and Methods section) in the Y-maze during a 5-minute trial. Values are expressed as median ± S.E.M. and representative of n = 4 different experiments involving 6–9 mice per group. ** *P*<0.05 indicates significant values compared with wild-type (WT) control mice (Mann–Whitney *U*-test).

To investigate whether reduced anxiety of *Fpr2/3* ^-/-^ mice was linked to an increased preference for novelty, indicative of low anxiety, we next assessed the performance of animals on a novel object recognition task. This test has been widely used as an explicit test of novel versus familiar object discrimination and relies on the idea that animals tend to preferentially approach novel objects [48]. We found that *Fpr2/3* ^-/-^ mice and controls showed no difference in their exploration of two identical objects (**Fig. 5A**, left panel). However, following the introduction of the novel object, wild-type mice spent about 40% of their time with the novel object as previously reported [49], [50] while *Fpr2/3* ^-/-^ mice spent a significantly greater proportion (about 60%) (**Fig. 5A**, right panel).

### *Fpr2/3* ^-/-^ mice show no difference in the Y-maze test

We next tested the *Fpr2/3* ^-/-^ mice in the Y maze. In this test mice tend to enter the maze arm that was explored most recently and recall the order of the arm entry. As shown in **Fig. 5B**, there were no significant difference between wild types and *Fpr2/3* ^-/-^ mice in the number of arm entries or percentages of alternations in this maze. These data show that *Fpr2/3* ^-/-^ mice are not impaired on a spatial memory task and imply that the effects reported earlier pertain mainly to diminished anxiety and fear-related responses in this group of animals.

### Higher basal corticosterone levels in *Fpr2/3* null mice

**Figure 6:**
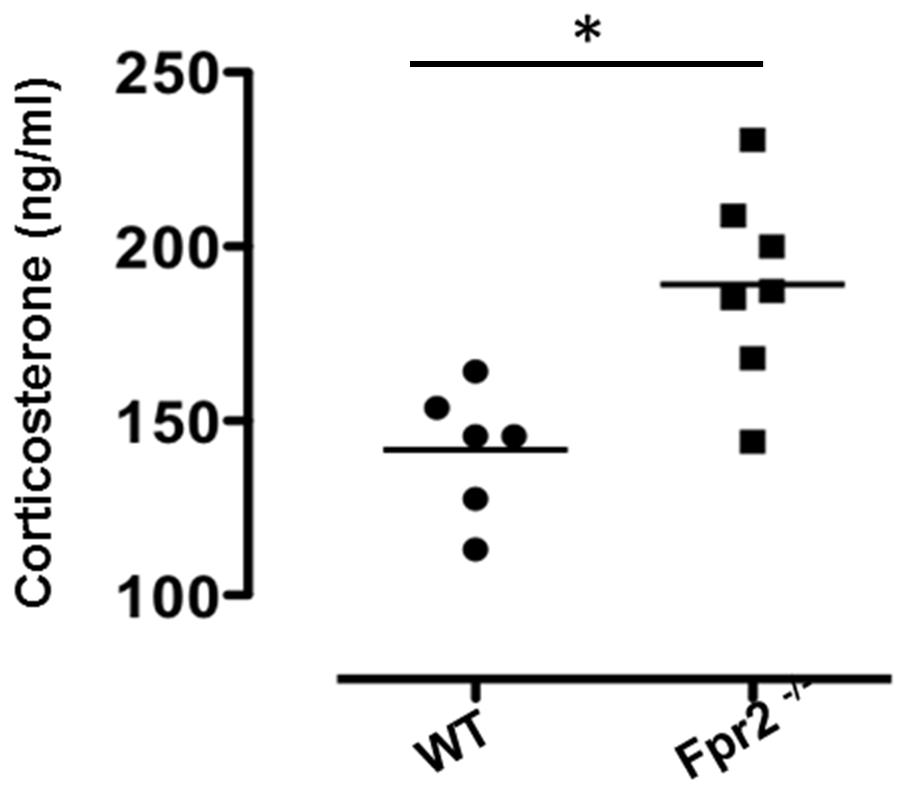
Increased level of corticosterone in *Fpr2/3* ^-/-^ mice. Levels of corticosterone in the plasma of WT and *Fpr2/3* ^-/-^. Values are expressed as ngml^−1^ and are representative of three experiments with 6 mice.

To determine whether the apparent differences in behaviour we observed were due to latent infection or inflammation, we performed a number of biochemical tests on serum samples. We found no significant differences in 12 inflammatory cytokines between wild-type and *Fpr2/3^-/-^* mice (data not shown). However, levels of circulating corticosterone were markedly higher in *Fpr2/3^-/-^* mice compared with controls (**Fig. 6**). These data are consistent with other findings showing a positive correlation between high responsiveness in a novel environment and hypothalamic-pituitary-adrenal axis activation [58]–[60].

### Administration of an FPR antagonist reduces some anxiety behaviours

**Figure 7:**
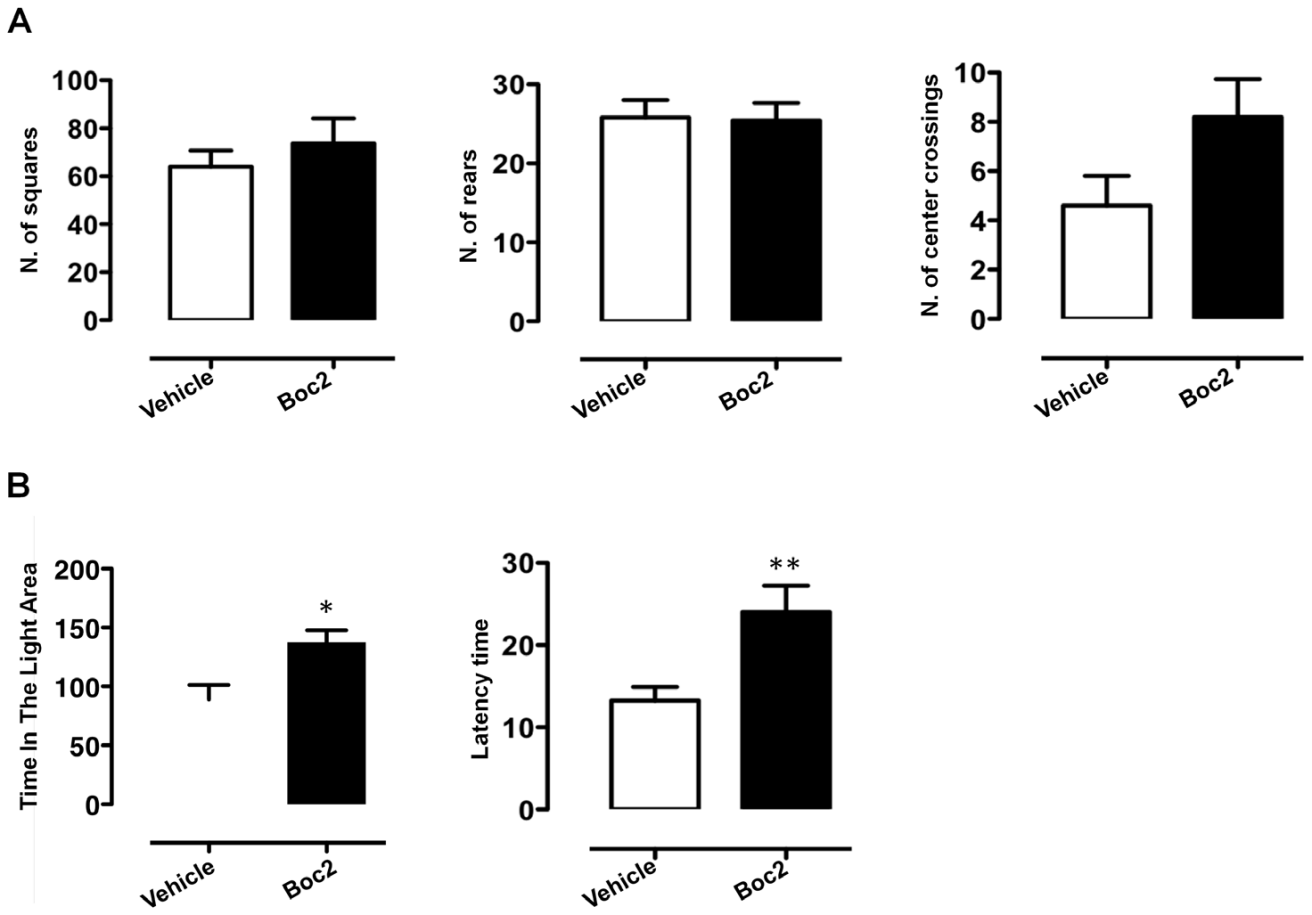
Boc2-treatment reduces anxiety-like behaviour in C57BL/6 mice. The bar graphs in **A** show the total number of squares crossed, rears and centre crossings of Boc2-treated mice compared to PBS vehicle-treated during a 5-minute trial in the open field test. The bar graph in B shows the total time (seconds) spent in the lit area and the latency (seconds) to first cross to the dark chamber of Boc2-treated mice compared to PBS vehicle-treated mice during a 5-minute trial. Values are expressed as median ± S.E.M. and representative of four different experiments involving 6 mice per group. * *P*<0.05 and ** *P*<0.01 indicate significant values compared to PBS-vehicle treated mice (Mann–Whitney *U*-test).

**Figure 8:**
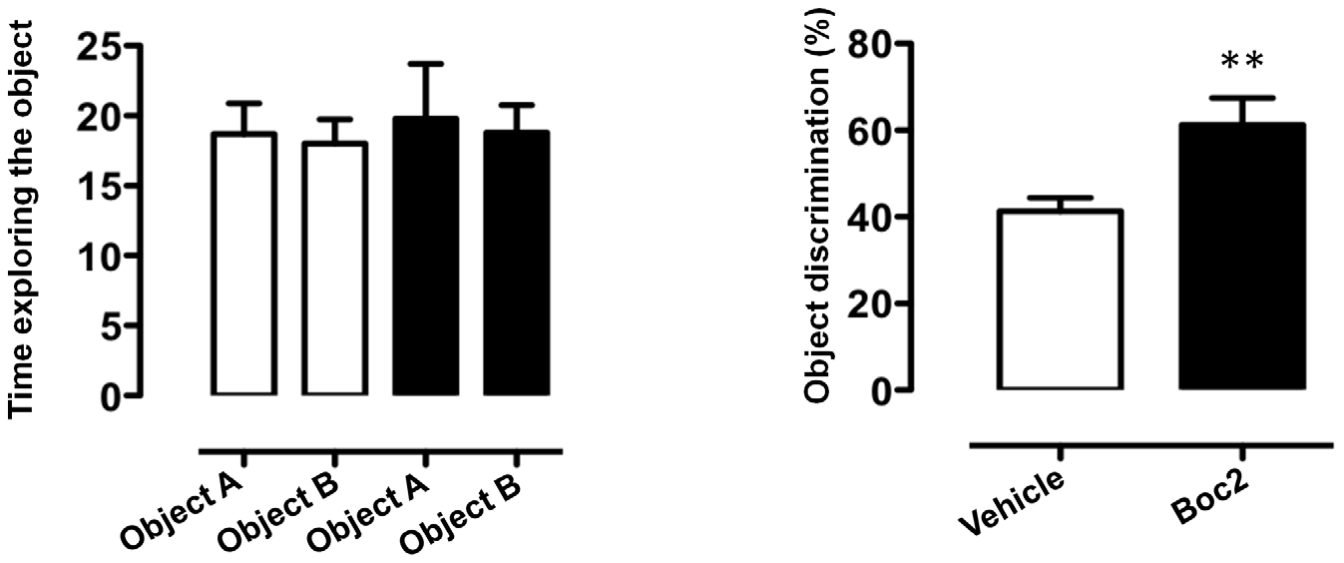
Boc2-treatment increases the recognition of a novel object in C57BL/6 mice. The bar graphs show the total time (seconds) spent exploring the objects used in the test during the 10-minute acquisition phase (left panel) and the % of time spent on the novel object in the subsequent 5-minute test phase (right panel) of Boc2-treated mice compared to PBS vehicle-treated mice. Values are expressed as median ± S.E.M. and representative of four different experiments involving 6 mice per group. ** *P*<0.01 indicates significant values compared to PBS-vehicle treated mice (Mann–Whitney *U*-test).

We next investigated whether the reduced anxiety of *Fpr2/3* ^-/-^ mice could be mimicked by administering the FPR inhibitor Boc2 in wild type animals. As shown in **Fig. 7**, Boc2 had no significant effect on general locomotion or explorative behaviour in the open field test (**A**) but did increase both the time in the brightly lit aversive compartment and the latency to cross to the ‘safe’ dark compartment (**B**). Moreover, Boc2-treated mice showed an increased preference for the novel object on the object recognition task compared with vehicle-treated wild-type animals (**Fig. 8**). These findings suggest FPR blockers may reduce some anxiety-related behaviours, including neophobia

### Reduced FPR signalling in the gut of *Fpr2/3* ^-/-^ and Boc2-treated mice

**Figure 9:**
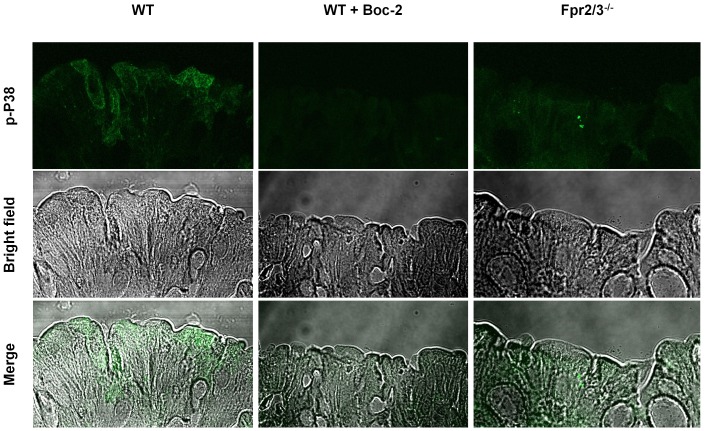
Reduced p-p38 staining in the colon of Boc2-treated C57BL/6 and *Fpr2/3* ^-/-^ mice. Immunofluorescence (top panel) of phospho-p38 of intestinal whole mount preparations (as described in Material and Methods) in either PBS-vehicle treated, Boc2 treated or *Fpr2/3* ^-/-^ intestinal mucosa. The middle and bottom panel show the bright field and the overlay pictures of the same samples.

Since the behavioural phenotype of *Fpr2/3* ^-/-^ mice could be partly reproduced by FPR antagonism we next investigated whether these effects were related to changes in the local (peritoneal) microenvironment. Given previous findings showing a key role of FPRs in regulating gut microbiota homeostasis we measured p-p38, a widely recognised intracellular readout of FPR activation [61]. Fluorescence microscopy of colonic tissue confirmed our prediction of increased staining for p-p38 in wild-type mice compared with *Fpr2/3* ^-/-^ and Boc2-treated mice (**Fig. 9** **)**.

## Discussion

The results of this study suggest that genetic deletion of *Fpr2/3* in mice causes significant changes in anxiety-related behaviour. Our experiments expand on previous observations made by Gao *et al.* on the behaviour of *Fpr*1^-/-^ mice [30] in terms of exploratory activity, anxiety, and fear-associated memory. The present study confirms and extends these findings by revealing reduced anxiety of *Fpr2/3* ^-/-^ mice on a range of tests of anxiety, including open-field and climbing exploratory behaviour, choice preference for aversive versus non-aversive contexts, and novel versus familiar objects. However, loss of *Fpr2/3*function did not affect species-specific activities such as burrowing or nest construction (data not shown). We also found a significant increase in the level of corticosterone in *Fpr2/3* ^-/-^ mice compared with controls. This is consistent with previous studies suggesting that altered baseline concentrations of cortisol in blood plasma is one of the features of anxiety disorders [62]. Often considered a biomarker of stress [63]–[65], the level of corticosterone does not always correlate with the level of anxiety. Indeed, studies for instance on the anxiolytic effects of enriched environment or voluntary exercise in experimental animals have reported conflicting data including no changes in corticosterone [66], [67] or an initial increase followed by a decrease to basal levels [68] or, as in our case, a significant increase [69]–[71]. We were very intrigued by the results of these studies since, as for the enriched or ‘exercised’ mice, the *Fpr2/3* ^-/-^mice show both increased level of corticosterone and an overall increase in exploratory and locomotory activity as shown by the open field (Fig. 1) and climbing test (Fig. 2). In light of these findings it is tempting to speculate that the increased corticosterone levels in *Fpr2/3* ^-/-^ mice might be the results of their increased ‘engagement’ with the external and social environment. Interestingly, these data contrast with those observed in *Fpr1* ^-/-^ mice [30] suggesting orthogonal regulation of corticosterone levels by FPR2/3 and FPR1 receptors.

Further exploration of the inquisitive and fearless nature of *Fpr2/3* ^-/-^ mice using the novel object test showed an almost 50% increase their discriminatory activity and no difference in spatial memory. These results suggest that the absence of homeostatic FPR2/3 signalling might induce a state of behavioural disinhibition and reduced anxiety. This conclusion is consistent with the widely recognised sensing/alerting function of FPRs in the olfactory system [29] and thus provides a further example of behavioural modulation by FPR signalling.

To support this hypothesis and to explore the therapeutic potential of our findings we investigated the effects of a FPR blocker on behaviour. Our findings reveal that administration of the pan-FPR antagonist Boc2 induced a behavioural profile that resembled, at least in part, what we observed in the *Fpr2/3* ^-/-^ mice. We think that this is most likely due to the metabolism of this inhibitor and hence to its transient effect as previously shown [72], [73]. Thus, we observed a significant increase in the number of center crossings in the open field and a significant increase in the time spent in the lit area of the light and dark box, both observations that are indicative of reduced anxiety. We also observed a marked improvement in the ability of wild-type animals to discriminate the novel object. We found that these differences (readily detectable after as little as 2 hour post treatment) were present only after intraperitoneal but not intravenous administration of Boc2 (data not shown). The lack of effect of intravenous administration of Boc2 led us to test whether Boc2 inhibited FPR signalling in the gut or intestinal mucosa.

A number of studies have shown that the intestinal mucosa expresses receptors for formylated peptides produced by the gut microbiota [18]. These commensal bacteria are known to play important and non-detrimental roles for the host [74]–[76] and have provided a perfect example of consensual interaction between microbes and immune sentinels present throughout the gut. These immune-microbiome interactions are known to be an important part of a dual circuit that controls behaviour and overall emotional wellbeing [77]–[79]. Indeed, one of the best examples of this system are the germ-free mice that are known to show signs of increased anxiety and reduced neurogenesis [80]–[83].

**Figure 10:**
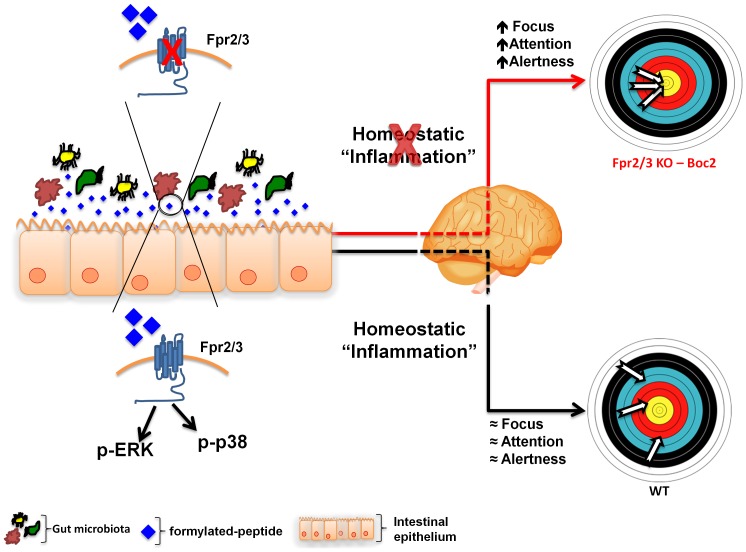
Hypothetical schema of the role of Fpr2/3 at the interface of the gut-brain axis. Non-pathogenic gut microbiota releases physiological levels of formylated peptides that activate FPR signalling in the gut epithelium. This homeostatic level of protective inflammation influences brain function maintaining a physiological level of focus and attention. The blockage of FPR signalling by an antagonist or the absence of gut microbiota causes a reduction in FPR activation and a parallel increase in the state of alertness, as observed in *Fpr2/3* ^-/-^ and Boc2-treated mice.

Our findings also show that both *Fpr2/3* ^-/-^ mice and Boc2-treated mice have a reduced immunostaining for p-p38 – a key FPR signalling pathway [4], [11], [84], [85]. Similar findings have been previously reported in other studies where it has been shown that commensal bacteria such as the Lactobacillus species stimulated these pathways in gut epithelial cells [19], [52], [74], [76]. It was recently suggested that the expression of FPR2 on the apical and lateral membrane of mouse colonic epithelial cells may have important biological significance, as it enables the epithelial cells to respond to both locally and systemically available ligands under various pathophysiological conditions [86]. Although we have not systematically explored this idea using a wider range of doses and other FPR antagonists our results show that the effects of Boc2 on behaviour occurs in parallel with a modulation of microbiota-induced FPR signalling in the gut. More specifically, the homeostatic and protective inflammatory state of the gut sustained by the commensal microbiota might contribute to a “homeostatic” status of focus and alertness that feature what we know as physical and mental wellbeing. Conversely, in the absence of this physiological loop a state of alertness and reduced anxiety might help the host to “focus” on the possible origin of “internal conflicts and dangers” (**Fig. 10**).

The validation of this model would have a significant translational impact for a variety of disorders that express impaired levels of attention and focus and a strong anxiety component, including obsessive compulsive disorder (OCD). Indeed, a number of recent studies have shown that dysfunctions of the gastrointestinal and immune systems are common comorbidities of anxiety related disorders [87]–[90]. Therefore, modulation of the microbiota through administration of FPR antagonists or genetically-engineered probiotic bacteria releasing Boc2-like peptides might represent a novel strategy for the treatment of a number of cognitive and anxiety-related brain disorders.
